# Brigham Hall, Canandaigua, N. Y.

**Published:** 1857-01

**Authors:** 


### EDITORIAL DEPARTMENT.

---

*Brigham Hall, Canandaigua, N. Y.*—We are in the habit of speaking of the provision for the insane in our State as to some extent ample and satisfactory. The immense asylum built at Utica, has always been fortunate in the character of its medical officers and management, and has sustained a high reputation as a noble charity. But out of this very excellence has grown an evil. Extensive in its accommodations, it has been regarded as sufficient, while our poor-houses are packed with insane men and women consigned to hopeless incurability. Conducted by medical officers of acknowledged skill and humanity, it has been erroneously supposed that its medical management approached perfection.

So far as quality is concerned, we have not a word of fault to find with the management of the Utica Asylum, but in respect to quantity it is but too evident that there is a great deficiency. We have somewhere mislaid the last Report of the Utica Asylum, and can only state in general terms that each physician attached to the institution has under his care some two hundred patients, each of whom should be seen daily, and some of them much more frequently. Aside from the actual time consumed in personal attention on patients, the physicians have laborious records to keep up and a large correspondence with the friends of their patients.

That under this pressure of labor the physicians are enabled to accomplish so much and make their institution a great blessing to community, is a meed of praise which we cheerfully accord to them, but not to the system.

The State Asylum is a public institution. Maintained as a charity by taxation of the people, its managers feel bound to prefer those patients who are, from poverty, most in need of assistance. The principle is a good one, but it results in a curious anomaly. The wealthy patient is unable to secure the same skill which is accorded to the poor. In the hundreds of applications for admission which are yearly rejected for want of room, it is the rich, and not the poor, who are sent away.

Neither have we other institutions in the State capable of affording shelter to this large class of applicants. The Bloomingdale Asylum labors under the same difficulties as that at Utica, and "Sanford Hall," a private institution of excellent character, located at Flushing, L. I., is only able to accommodate the wealthy class from its immediate vicinity.

There is, then, a serious want of accommodations for the insane in our State. Government will soon be compelled to enlarge the provisions for the insane poor, but it will always be difficult to provide satisfactorily for the rich by any public institution.

With a view to the remedy of these evils, and at the same to provide an attractive field of usefulness for himself, Dr. George Cook, for many years one of the medical officers of the Utica Asylum, has opened an institution at Canandaigua, which he has named, in honor of the lamented Dr. Brigham, "Brigham Hall."

It was our privilege to spend a Sunday beneath its roof a short time since, and it affords us pleasure to testify to its excellence in every department. Dr. Cook resides at the "Hall," and our friend, Dr. Edson Carr, is the consulting physician.

"Brigham Hall" is located in a grove on a hill near the village of Canandaigua, commanding a lovely view of the lake and of a charming rural scenery. The house has no resemblance to an asylum, but looks like an extensive yet quiet family residence. It has been constructed with great care. Sewerage, warmth and ventilation, are all provided for, and the conveniences for bathing and exercise, are all that can be required.

It has no proper wards, but is cut up into numerous well lighted pleasant rooms, opening on wide cheerful halls. This is a system requiring more numerous attendants and greater expense, than any other, but it has corresponding advantages. The rooms are all neatly furnished, the walls papered, the floors carpeted, and have a bright cheerful home-like aspect, which cannot fail to exert a salutary influence on the diseased mind. The entire house is lighted by gas manufactured on the premises.

It affords us much pleasure to be able to speak from personal knowledge of the excellencies of this institution, and to recommend it to those physicians who have patients requiring such a residence. We should not omit to mention that Dr. Cook also devotes much attention to nervous diseases not involving the mind. All medical men occasionally feel the want of the necessary conveniences for treating this class of patients, and will be glad to learn that there is a legitimate place to which they may be sent instead of consigning them to the chances of water-cures.

Applications for the admission of patients, accompanied by a brief history of the case, and the condition of the patient at the time the application is made, should be addressed to Dr. Cook. The terms will depend upon, and vary with, the amount of care and attention that may be required.

One of the great sorrows of insanity is the necessity of removal from the tender care of immediate friends, and the greatest alleviation of this affliction is found in the knowledge that the poor victim of disordered mind may find kind and careful treatment, and be surrounded in his seclusion by the comforts of home.

